# Wakȟáŋyeža (Little Holy One) - an intergenerational intervention for Native American parents and children: a protocol for a randomized controlled trial with embedded single-case experimental design 

**DOI:** 10.1186/s12889-021-12272-9

**Published:** 2021-12-18

**Authors:** Teresa Brockie, Emily E. Haroz, Katie E. Nelson, Mary Cwik, Ellie Decker, Adriann Ricker, Shea Littlepage, Justin Mayhew, Deborah Wilson, Lawrence Wetsit, Allison Barlow

**Affiliations:** 1 Johns Hopkins School of Nursing, 525 N. Wolfe St, Baltimore, MD 212105 USA; 2 Johns Hopkins Bloomberg School of Public Health, Center for American Indian Health, 415 N. Washington St. 4th Floor, Baltimore, MD 21231 USA; 3 Fort Peck Community, 121 East Indian Street Wolfe Point, Montana, 59201 USA

**Keywords:** Childhood trauma, Native American, Parenting, Youth, Intergenerational intervention, Randomized control trial, Single-case experimental design

## Abstract

**Background:**

Trauma within Native American communities compromises parents’ parenting capacity; thus, increasing childrens’ risk for substance use and suicide over the lifespan. The objective of this manuscript is to describe the Wakȟáŋyeža (Little Holy One) intervention and evaluation protocol, that is designed to break cycles of intergenerational trauma, suicide, and substance use among Fort Peck Assiniboine and Sioux parents and their children.

**Methods:**

A randomized controlled trial with an embedded single-case experimental design will be used to determine effectiveness of the modular prevention intervention on parent-child outcomes and the added impact of unique cultural lesson-components. Participants include 1) Fort Peck Assiniboine and Sioux parents who have had adverse childhood experiences, and 2) their children (3–5 years). Parent-child dyads are randomized (1:1) to Little Holy One or a control group that consists of 12 lessons taught by Indigenous community health workers. Lessons were developed from elements of 1) the *Common Elements Treatment Approach* and *Family Spirit,* both evidence-based interventions, and 2) newly created cultural (intervention) and nutrition (control group only) lessons. Primary outcomes are parent (primary caregiver) trauma symptoms and stress. Secondary outcomes include: Parent depression symptoms, parenting practices, parental control, family routines, substance use, historical loss, communal mastery, tribal identity, historical trauma. Child outcomes include, externalizing and internalizing behavior and school attendance. Primary analysis will follow an intent-to-treat approach, and secondary analysis will include examination of change trajectories to determine impact of cultural lessons and exploration of overall effect moderation by age and gender of child and type of caregiver (e.g., parent, grandparent).

**Discussion:**

Many Native American parents have endured adverse childhood experiences and traumas that can negatively impact capacity for positive parenting. Study results will provide insights about the potential of a culturally-based intervention to reduce parental distress – an upstream approach to reducing risk for childrens’ later substance misuse and suicidality. Intervention design features, including use of community health workers, cultural grounding, and administration in Head Start settings lend potential for feasibility, acceptability, sustainability, and scalability.

**Trial registration:**

ClinicalTrials.gov: NCT04201184. Registered 11 December 2019.

## Introduction

### Background and rationale

There is strong evidence that negative parenting experiences in early childhood are associated with later adolescent and young adult substance use and suicide [1, 2]. The earliest effects of ineffective parenting can be observed in early childhood (0–5 years old), expressed as externalizing and internalizing behaviors, such as aggression and social withdrawal [3–5]. Externalizing and internalizing behaviors in early childhood predicts poor school performance in the middle years, and drug use, anti-social, delinquent, and aggressive behavior, and risk of suicide in middle and late adolescence [4, 6–9]. Early parent behaviors most associated with externalizing behaviors in early childhood include coercive interactions, poor monitoring, and harsh, unresponsive, or rejecting parenting [4, 10, 11]. Parenting styles that have been linked to internalizing disorders include negative criticism, hostility, over-control, and abuse or neglect [5]. Parenting factors associated with childrens’ future substance use and suicide risk include parental stress, alcohol and drug use, parent-child attachment, multigenerational adversity, and poor monitoring [9, 10, 12–14]. Past research has shown high numbers of adverse childhood experiences (ACEs) negatively impact parents’ ability to optimally raise their children. Adults who experience ACEs are at increased risk of depression, substance abuse, and antisocial behavior—risk factors for poor child-rearing practices [15, 16]. ACEs in parents have been linked to high parenting stress, poor parent-child attachment, and harmful parenting practices such as corporal punishment during infancy [17–20]. These trends in parents create a cycle of intergenerational ACEs, whereby children of parents who experience ACEs are at higher risk for ACEs themselves and grow up at risk for perpetuating negative parenting practices [2, 21].

Among all United States (U.S.) populations, Native Americans report the highest ACE scores [1, 22, 23] At a population level, ACEs have a strong dose-response impact, with more ACEs contributing to worse lifetime health outcomes, including increased risk for alcoholism, drug abuse, depression, and suicide attempts [19]. This relationship has also been documented in the participating Fort Peck community. Our prior research with *N* = 288 youth ages 15–24 years old found 96% of the sample reported at least one ACE, 3.5 times higher than other populations (26%) [24]. Additionally, two ACEs were reported by 75% of youth, almost twice that of other studies (40%) [1]. After controlling for age, gender, tribal affiliation, and school attendance, each additional ACE increased the odds of PTSD (60%), depression (30%), suicide attempt (36%), and polydrug use (73%) [1, 24]. To break the cycle of ACEs and negative parenting, early parenting interventions that address risk factors for ineffective parenting through direct training are needed [24–27]. Delivering these types of interventions during developmental transition time periods, such as when children are moving from home settings to their first school days, may be particularly impactful [9].

### Objectives

Accordingly, the objective of this trial is to evaluate the Little Holy One intervention’s impact on known family-based risk and protective factors for youth suicide and substance use, starting in early life, through three specific aims. Embedding community-identified strengths of the Fort Peck Assiniboine and Sioux Tribes, the intervention is designed to: 1) reduce parents stress and trauma-related symptoms using culturally adapted components of the *Common Elements Teaching Approach (CETA*), 2) improve parenting skills (monitoring and nurturing), using components of *Family Spirit* that was designed by Native Americans for use with Native American parents and children, and 3) promote positive tribal identity and communal mastery, and address historical and contemporary trauma, factors found to be protective factors for high-risk substance use and suicide within the participating community, by using cultural components that incorporate traditional practices designed by cultural leaders in the community [1, 28–33].

*Aim 1: To evaluate the effectiveness of community health worker delivered Little Holy One on parent and child behavioral and mental health outcomes, using a randomized controlled trial design.* We hypothesize that a) parents in Little Holy One will experience significant decreases in parenting stress and trauma-related symptoms, and significant increases in positive parenting behavior, tribal identity, communal mastery, and b) their children will have lower levels of externalizing and internalizing behavior compared to children in the active control group condition. *Aim 2: To explore the benefit of specific cultural enhancements (promoting tribal identity and communal mastery) of the intervention on parents’ mental health and well-being, by using a single-case experimental design embedded within the randomized controlled trial*. We hypothesize that specific cultural components will increase the impact of Little Holy One by providing immediate and sustained reductions in parental stress after component completion. *Aim 3: Explore how the social network characteristics (*i.e.*, network size, average strength of ties, heterogeneity, density, % same race, % same age, % same gender) of Native American parents are related to both risk for and protection from suicide and opioid use*. Using ego network data, we will extract information about each respondent’s unique network characteristics as correlates of suicide and opioid. *Aim 3a: Examine the impact of the Little Holy One family-based prevention program on the social networks of Native American parents.* We will use pre- and post-intervention data to explore the effect of the intervention on parents’ social networks. *Aim 3b: Determine whether program effects on social networks mediate effects on suicide and opioid among parents*. The effectiveness of behavioral interventions often occur through multiple strategies, the strategies presented in the parent grant remain unchanged, however, we aim to explore the possible efficacy of the Little Holy One program on social networks as levers of change for conferring reduced risk of suicide and opioid use as the group-based nature of the intervention increase social interaction of parents, increases parenting support networks, and is founded in similar values of cultural strengths.

## Methods

### Conceptual model

**Fig. 1:**
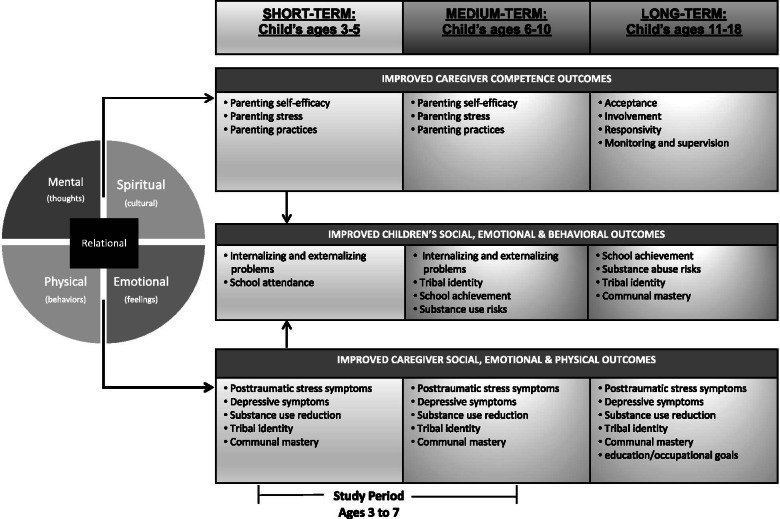
Little Holy One conceptual model

Our conceptual model (Fig. 1), developed in partnership with our participating communities, drove selection of the three core intervention components: 1) Elements of *CETA* will be applied to reduce parents’ stress and trauma-related symptoms. 2) Elements of *Family Spirit* will be applied to improve parenting. 3) Cultural components will promote tribal-specific protective factors, including positive tribal identity and communal mastery while buffering the effects of historical trauma [27, 31, 34–37]. The curriculum is designed to be strengths-based and holistic, promoting family wellness across mental (cognitive), physical (behavioral), emotional (feelings), and spiritual domains represented as balanced quadrants in the Native American Medicine Wheel. Further, both the relational nature of the cultural teachings and the employment of culturally embedded community health workers to teach the curriculum are intentional design choices aimed at strengthening relational ties between parents and children, families and community health workers, and families and the community. This strategic approach responds to the growing literature about the important role that connectivity plays in prevention of suicide risk among Native Americans [32, 33]. Little Holy One is designed to impact modifiable components of parenting, mental health, and culture to reduce early childhood risk for behavioral health outcomes.

### Trial design

This is a randomized controlled trial (RCT) with 1:1 randomization of 120 parent-child dyads to Little Holy One or an active control group. Within the RCT there is an embedded single-case experimental design (SCED) aimed at exploring the added impact of specific cultural components [38].

### Study setting

The study is taking place on the 2-million-acre Fort Peck Reservation. The Fort Peck Reservation was established by treaty in 1888, in rural northeastern Montana, and home to ~ 13,000 Dakota and Nakoda members, 50% of whom are < 18 years [34, 35, 39]. The reservation lies within one of the poorest and least healthy counties in the United States (U.S.) [2, 31]. In 2011, the violent crime rate was five times the state and three times the U.S. rate. Health and safety are the highest priority concerns for the tribal nation [34, 35, 39].

### Participants

Participants are parents and their children ages 3–5 years old, who are enrolled in Fort Peck Head Start (*N* = 120 dyads). Intervention and data collection will be completed in private locations, convenient for participants, including Head Start centers, study offices, and homes in the reservation communities of Poplar and Wolf Point. Head Start is a federal program run through the U.S. Department of Health and Human Services intended to prepare children from low-income families for school [40]. Two of the four Head Start centers on the reservation are participating, as they primarily serve children ages 3–5 years old. The Fort Peck Head Start Program averages about 237 students ages 3–5 years old per year; approximately 80% live below the 100% poverty level, and 40% are from single-parent families. In 2015, the parent age distribution was 12–24 years (16%), 26–35 years (66%), and 35–45 years (18%).

### Eligibility criteria

To be included in the study a participant must meet the following criteria: 1) parent that is ≥18 years old with a child 3–5 years old enrolled in participating schools (Poplar or Wolf Point); 2) willingness to participate in all aspects of the study including random assignment; 3) parent has experienced at least one ACE, and recent or historical trauma; and 4) the child is an enrolled member or the descendant of an enrolled member of the Fort Peck Tribes. For the purposes of this study, parents are being defined as the primary caregiver of the child. Participants will be excluded if they indicate they are planning to move or know they cannot otherwise take part in the full intervention during baseline enrollment.

### Recruitment and consent/assent

Participants will be recruited through referrals from Head Start staff and through flyers and study team attendance at Head Start related events. Potential participants who express interest will be provided with a brief overview of the study and complete a phone screen to determine eligibility. Only one child per family is eligible – if more than one child in a family meets eligibility criteria, we will randomly select which child will be included in the study. If participants meet eligibility criteria, study staff will schedule a time to complete consent and baseline assessment procedures. The parent will complete the consent form for their own participation. The parent or legal guardian of the child will also provide assent for the child to participate.

### Intervention

The Little Holy One intervention combines elements adapted from *CETA* (four lessons) and *Family Spirit* (four lessons) with four cultural lessons addressing tribal-specific risk (historical trauma) and protective factors (cultural identity, communal mastery) (Table 1).

**Table 1:** Little Holy One lessons and origin

Component	Origin	Participant(s)
Psychoeducation	*CETA*	Parent
Substance Use Prevention	*CETA*	Parent
Coping with Traumatic Stress Symptoms	*CETA*	Parent
Cognitive Coping Skills	*CETA*	Parent
Knowing Your Child	*Family Spirit*	Parent
Monitoring Your Child	*Family Spirit*	Parent
Daily Routines	*Family Spirit*	Parent
Skills for Healthy Living	*Family Spirit*	Parent
Promoting Tribal Identity	Cultural	Parent + Child
Enhancing Communal Mastery	Cultural	Parent + Child
Promoting Smudging	Cultural	Parent + Child
Healing Historical & Contemporary Trauma	Cultural	Parent

*CETA* was selected as the basis for Little Holy One for its proven ability to address stress-related problems in trauma-affected, low-resource communities [41–44]. *CETA* is originally a mental health treatment program, but we selected and adapted four *CETA* lessons and designed them to focus on prevention of mental health disorders rather than treatment. The lessons include a focus on 1) psychoeducation (Understanding Our Emotions), 2) cognitive coping skills (Thinking Positively), 3) substance use prevention (Alcohol and Drug Use), and 4) coping with traumatic stress symptoms (The Power of Our Memories) [45].

*Family Spirit* is an evidence-based intervention proven to promote positive parenting and childrens’ early social, emotional, and behavioral well-being among tribal communities [34, 46]. Original lessons were taught to parents with children ages 1–3 years but were adapted for parents of children ages 3–5 years for Little Holy One. Lesson themes include: 1) understanding children’s emotional, social, and physical development; 2) “mindful parenting”—focused on nurturing monitoring and supervision; 3) avoiding power struggles that lead to childrens’ behavioral problems by establishing healthy, consistent routines; and 4) “skills for healthy living” that promotes healthy family relationships and planning for childrens’ futures.

Four cultural lessons, developed in partnership with the Tribal Advisory Board and key community leaders, are designed to 1) promote tribal identity, 2) promote communal mastery (strengthen family and community), 3) heal historical and contemporary trauma, and 4) promote smudging as a family-based spiritual practice. Parents are receiving all 12 lessons, while children participate with their parents in three of four cultural lessons on tribal identity, communal mastery, and smudging. Children are being excluded from the fourth cultural lesson given the sensitive subject matter, and it will allow parents the space to explore historical and contemporary trauma without having to tend to their child’s needs. Each of the 12 intervention lessons are designed to be approximately one to one and a half hours in length.

### Control condition

The active control condition has interventionists that teach nutrition education topics (Table 2**)** in six group sessions of up to 7–8 parent-child dyads per group. Parents learn about healthy food options, where to access healthy foods, are given healthy recipes, and sample easy-to-make healthy foods with their children. The nutrition lessons are approximately 1 hour in length. Control content was selected by the Tribal Advisory Board based on its potential benefit and interest to participating families, with minimal risk for contaminating intervention outcomes.

**Table 2:** Lesson schedule for all study participants

Week	Little Holy One Lessons (Intervention)	Nutrition Lessons (Control)
1	**Ą́**ba Wasté / Aŋpétu Wašté (Promoting Tribal Identity)	Racing Towards Healthy Eating
2	Understanding our Emotions	–
3	Azí’įc’iya / Azídya (Promoting Smudging)	Fun with Food Groups!
4	Thinking Positively	–
5	Encouraging Early Learning	Portion Sizes
6	Daily Routines for Caregivers and their Children	–
7	Alcohol and Drug Use	Making Your Plate
8	Monitoring your Child’s Routine	–
9	Strengthening Family and Community (Enhancing Communal Mastery)	Rethink Your Drink!
10	The Power of Our Memories	–
11	Healing Historical Trauma	Savor It: Smell, See, Taste!
12	Skills for Effective Caregiving	–

### Lesson frequency and duration

**Fig. 2:**
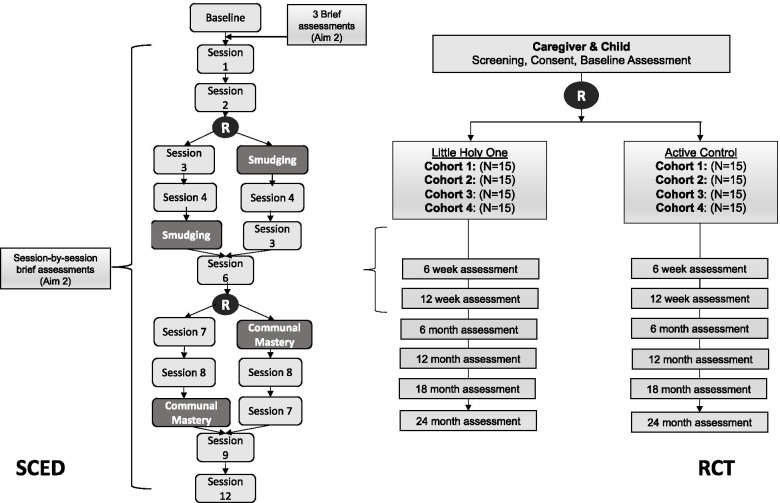
Overall study flow and SCED

Participants in the intervention group will be taught the 12 lessons over a 16-week period in individual sessions with their community health worker—a delivery strategy selected to enhance participant engagement, local acceptability, and sustainability [47, 48]. Lessons will be delivered at a location that works best for the participant, including Head Start locations, community centers, or study office space at a convenient time (i.e., after drop-off or pick-up). Participants in the control group will participate in group or individual lessons on nutrition at a convenient time and place for participants. Both the intervention and control lessons will begin 2 weeks after study enrollment, and baseline assessments and randomizations have been completed. Participants in both conditions will be assessed by blinded evaluators using mobile data collection at baseline, 12 weeks (when intervention ends), and 6-, 12-, 18-, and 24-months follow-up. Figure 2 outlines the overall flow of the study below.

### Randomization process and study flow

Participants are being recruited from Poplar and Wolf Point Head Starts in four cohorts over 2 years. Two cohorts of 15 dyads will be randomized each semester to the intervention or control conditions, for a total of 30 per intervention and 30 per control each year for 2 years. Within the Little Holy One intervention, participants will be further randomized at two time points: 1) after the second lesson participants will be randomized to immediately receive a smudging lesson in session 3 or wait until session 5 to receive the smudging lesson; and 2) after the sixth session, participants will either immediately receive the communal mastery lesson in session 7 or wait until session 9 to receive the communal mastery session (Fig. 2). First-order randomization to either Little Holy One or the active control, is aimed at understanding intervention effectiveness, while the second-order randomization aims to understand the impact of specific cultural components by controlling for time participating in the intervention. Embedding the SCED in an RCT will allow for understanding how specific cultural components may provide immediate benefit and can inform future adaptation of the program if proven effective and scaled.

### Allocation, concealment, and implementation

The intervention allocation sequence will be generated using the “ralloc” command in Stata with 7 total strata (3 for age, 2 for gender, and 2 for site), a date-based randomization seed, and blocked randomization with blocks of varying size (8–16). Participants will be stratified by Head Start site, child gender, and child age to ensure equal groups of 3, 4, and 5 years old in each of the conditions. If more than one child in a family meets eligibility criteria, we will randomly select which child gets included in the study. We are unable to block randomize by parent gender because local feedback has suggested the majority are women.

Allocation will be handled by Research Electronic Data Capture (REDCap) electronic data capture tools hosted at Johns Hopkins University [49, 50]. The project data manager will generate the allocation tables describing the randomized order of allocation and upload them into REDCap’s randomization module, which will then implement all allocation. Once uploaded, REDCap will not allow the allocation tables to be accessed; thus, the allocation process will be concealed and protected from error. All evaluators will be blinded to participants’ study assignment and would only become unblinded if essential for management of the participant.

### Measures

Measures were selected for their strong psychometric properties and prior use and acceptance in Native American communities. Primary outcomes will be assessed at baseline (2 weeks), 6 weeks, 12 weeks, 6 months, 12 months, 12 months, 18 months, and 24 months. Table 3 outlines the detailed measurement schedule with corresponding time points for assessment. Assessment modalities include parents’ self-reports, and parents’ and teachers’ reports on children, allowing for rigorous analysis and triangulation of results. Measures are described by aim below.

**Table 3:** Outcome measures and assessment time points

	# of items	Baseline	Pre-session 1	Each session	6 weeks	12 weeks	6 months	12 months	18 months	24 months
**AIM 1**										
***CAREGIVER: Mental/Emotional***										
PTSD Checklist for DSM-5	13	x			x	x	x	x	x	x
Parenting Stress Index - Short Form	20	x			x	x	x	x	x	x
Stressful Life Events Questionnaire	13	x								
CESD-R-10	10	x				x		x		x
Benevolent Childhood Experiences	10	x								
Adverse Childhood Experiences	17	x								
***CAREGIVER: Physical***										
Parenting Practices Interview	72	x				x		x		x
Parental Locus of Control	47	x				x		x		x
Family Routines Index	28	x				x		x		x
WHO ASSIST	10	x				x		x		x
***CAREGIVER: Spiritual***										
Historical Trauma	3	x								
Historical Loss Scale	12	x								
Communal Mastery Scale	10	x				x		x		x
Orthogonal Cultural Identification Scale	6	x				x		x		x
Historical Trauma Checklist	16	x								
***CHILD: Mental/Emotional***										
Strengths and Difficulties Questionnaire	30	x					x	x		x
***CHILD: Physical***										
School Attendance	–	x			x	x	x	x	x	x
**AIM 2**										
Perceived Stress Scale	10	x	x	x		x	x	x	x	x
**AIM 3: SOCIAL NETWORKS SUPPLEMENT**										
Caregiver Ego Networks	17	x				x		x		x
Columbia Suicide Severity Rating Scale	6	x				x		x		x

***Aim 1:*** *To evaluate the effectiveness of community health worker delivered Little Holy One on parent and child behavioral and mental health outcomes, using a randomized controlled trial design.*

Primary outcomes – Parent:

1. Change in parent trauma symptoms will be self-reported at 2 weeks to 24 months using the *Post-Traumatic Stress Disorder (PTSD) Checklist for the Diagnostic and Statistical Manual of Mental Disorders (DSM) -5* (PTSD Checklist for DSM-5, Civilian Version) [51].
2. Change in caregiver parenting stress will be self-reported using the *Parenting Stress Index – Short Form* (PSI-SF). The PSI-SF is a 36-item self-report that measures three domains of parenting stress: parental distress, parent-child dysfunctional interaction, and difficulty with child [52]. Scores range from 36 to 180. Score ranges are represented as percentiles; 15–80 is considered a typical stress percentile, 81–89 is considered a high stress percentile, and 90–100 is considered a clinically significant stress percentile. The PSI-SF was used in previous studies with Native American populations and performed well [27, 31, 34, 35].

Secondary outcomes – Parent:

1. Change in parent depression symptoms will be measured by self-report with the *Center for Epidemiologic Studies Depression Scale-Revised* (CESD-R-10) [53]. The CESD-R-10 is comprised of 10 items based on DSM-IV diagnostic criteria for Major Depressive Disorder. Scores range of 0–30, with a score greater than eight indicate clinically significant symptoms. The CESD-R-10 is based on the CESD, a widely validated instrument, including among Native American populations [27, 34, 53, 54].
2. Stressful Life Events will be assessed using the *Stressful Life Events Screening Questionnaire* (SLESQ)**,** a 13-item self-report questionnaire designed to assess lifetime exposure to potentially traumatic events [55, 56]. It has been used in several culturally diverse settings and is recommended for use for research purposes [55].
3. Parents’ positive childhood experiences will be measured by self-report at baseline with the *Benevolent Childhood Experiences Scale* [57]. This is a 10-item scale designed to assess positive childhood experiences in adults with experience of mistreatment or adversity.
4. Parents’ ACEs will be measured by self-report at baseline with a 23-item ACEs scale adapted to the study population [58].
5. Parenting practices will be measured by the *Parenting Practices Interview* (PPI), a 72-item self-report questionnaire adapted from the Oregon Social Learning Center’s Discipline Questionnaire and revised for young children [59]. It measures the disciplinary style of a parent and has been used in a variety of settings and populations [60].
6. Parental control will be measured by the *Parental Locus of Control Scale* (PLOC), a 47-item questionnaire which measures five factors to assess the locus of control a parent or caregiver has over a child [9].
7. Family routines will be measured via self-report using the *Family Routines Index,* a 28-item questionnaire which measures 10 areas of family routines [61].
8. Parent substance use will be measured via self-report using an adapted version of the 15-item *WHO ASSIST Questionnaire* [62], which screens for problematic or risky substance use. A risk score is provided for each of the 10 substances included in the survey. The ASSIST is reliable, valid, flexible, comprehensive, and cross-culturally relevant having been validated with populations all over the world [62, 63].
9. Parent historical loss experiences will be assessed via self-report at baseline with the *Historical Loss Scale.* The scale quantifies 12 types of losses that Native American tribes might have experienced in the past, how often they are thought about in the present, and 12 different symptoms that they might have because of thinking about these losses [64]. This scale has been used in the previous study (α = .93) and in several Native American populations [65–67].
10. Parent communal mastery will be measured via self-report using the 10-item *Communal Mastery Scale* [68, 69], which was developed specifically for Native contexts using two commonly employed measures of mastery and self-efficacy and adapted to add more collectivist statements. This scale was successfully used in a previous study at Fort Peck (α = .85).
11. Parent tribal identity will be assessed using a modified version of the 6-item *Orthogonal Cultural Identification Scale.* The scale has been adapted for Assiniboine and Sioux tribal identity and was also used in a previous study on the Fort Peck Reservation (α = .90) [70, 71].
12. Parents’ experiences related to historical trauma will be measured by the *Historical Trauma Checklist*. This measure is a 15-item checklist, developed from focus group discussions with the Fort Peck Reservation [24]. Three questions are posed to determine relevant historical trauma experiences.

Secondary outcomes – Child:

1. The *Strengths and Difficulties Questionnaire* (SDQ) and impact supplement will measure childrens’ externalizing and internalizing behavior via parent report. The SDQ is a 30-item scale which measures 25 attributes on five scales: emotional symptoms, conduct problems, hyperactivity/inattention, peer relationship problems, and prosocial behavior [72].
2. Head Start school attendance will be tracked via teacher report on an ongoing basis for all children enrolled in the study.

***Aim 2****: To explore the benefit of specific cultural enhancements (promoting tribal identity and communal mastery) of the intervention on caregivers’ mental health and well-being, by using a single-case experimental design embedded within the randomized controlled trial*.

Outcomes:

1. Parent stress will be measured using the *Perceived Stress Scale* (PSS). The PSS is a 10-item scale that has been widely used and validated, including in Native American populations [73, 74].
2. Parent communal mastery will be measured using the same 10-item *Communal Mastery Scale* highlighted above [68, 69].
3. Parent tribal identity will be assessed using a modified version of the 6-item *Orthogonal Cultural Identification Scale* as highlighted above [70, 71].

***Aim 3****: Explore how the social network characteristics (*i.e.*, network size, average strength of ties, heterogeneity, density, % same race, % same age, % same gender) of American Indian parents/adult caregivers are related to both risk for and protection from suicide and opioid use*. ***Aim 3a****: Examine the impact of the Little Holy One family-based prevention program on the social networks of Native American parents/adult caregivers.****Aim 3b****: Determine whether program effects on social networks mediate effects on suicide and opioid among parents/caregivers*.

Outcomes:

1. Social networks information will be collected via a 17-item self-report *Caregiver Ego Networks Questionnaire.* Parents will provide information about tribal affiliation, relationships, substance use, communication, parenting support, and cultural involvement of up to 10 of their closest social relationships [75, 76].
2. Suicide risk will be measured with six items from the *Columbia Suicide Severity Rating Scale* (CSSR-S), which has been widely used to identify and assess individuals at risk for suicide across diverse communities and settings [77].

### Sample size and power

#### Aim 1

We based our primary sample size calculation on previous Family Spirit results indicating small to medium effect sizes for adult and child socio-emotional and behavioral outcomes 6 months post intervention completion [27, 31]. As such, power is based on detecting a small-medium difference between Little Holy One and control participants on caregiver and child outcomes 6-months post intervention completion (see below for power at 24 months post intervention). Since power analysis for mixed-effects regression models is complex, we use the approach described by Hox and Hedges [78, 79]. In this approach there are three steps: 1) estimate the power for a single-level regression model, ignoring clustering. This is considered the target sample size; 2) Compute the actual sample size for the proposed study that ignores the impact of clustering and accounts for attrition; and 3) Generate the effective sample size by penalizing the actual sample size with the design effect formula (i.e., n_eff_ = n / [1 + {n_clus_ – 1} ρ]). If the effective sample size is equal to or larger than the targeted sample size, then power is considered sufficient to detect the effect of interest. In our case, for step 1 power is .80 to detect a medium effect of f = .18 with 120 single-level, independent observations. For step 2, our actual sample size of 120 caregivers and children, with 5 measurement points, yields 600 (non-independent) observations. Accounting for attrition based on our previous work with family-based prevention interventions (i.e., the final *Family Spirit* trial had 18% attrition after 39 months’ intervention) [34], we used a conservative 30% attrition rate and adjusted our actual sample size to 420 non-independent observations. For step 3, using the design effect formula, accounting for 30% attrition and assuming a conservative nesting effect of ρ = 0.3 (to account for additional nesting within schools), our actual sample size of 420 non-independent observations provides the statistical power of 190 independent observations as our effective sample size. The effective sample size is more than the estimate for the targeted sample size indicating sufficient power to detect a small effect (f = .13). Sample size calculations for *all* aims can be found in Table 4**.**

**Table 4:** Sample size calculations

	N	α	Power (1-β)	Effect size (f)
**Aim 1**				
Parents	120	0.05	0.80	0.13
Children	120	0.05	0.80	0.13
**24-month outcomes**				
Parents	84	0.05	0.80	0.20
Children	84	0.05	0.80	0.20
**Aim 2**				
Parents	60	0.05	0.80	0.20
**Additional moderator exploration**				
Gender (Child outcomes)	60	0.05	0.80	0.24
Age (Child outcomes)	60	0.05	0.80	0.24
Type of caregiver (Parent and child outcomes)	60	0.05	0.80	0.24

#### 24-month outcomes

While not our primary outcome, based on our conceptual framework, effects on proximal outcomes should correspond to effects on more distal outcomes measured at 2 years post-intervention. As there are no other longitudinal follow-up intervention studies in Native American settings, we assume a very conservative 50% retention rate of our original enrolled sample. This will result in approximately *n* = 42 participants in each arm at *24-months post intervention* completion. With power set to 80% and alpha set to 0.05, with *n* = 42 participants in each arm, and an additional 2 assessment time points, we will be able to detect an average effect size of f = 0.20 (medium effect) on continuous outcome measures between the groups.

#### Aim 2

Using the same approach as our Aim 1 power calculation, we will be powered to detect a medium effect (f = 0.20) in level of perceived stress (PSS) following delivery of the cultural components.

#### Additional planned analysis

We will explore moderation of intervention effects by gender and age of the child, and type of caregiver. We are not measuring sex as a biological variable, so our analysis of sex differences is limited by a self-report gender of child indicated at baseline interview. While our aims are not powered to detect moderation effects, exploration of moderation in this RCT will serve as hypothesis generating and identify potential differing effects by gender and age to explore in future studies with larger sample sizes [80]. Moderators will be explored by adding a three-way interaction term to the models (e.g., Intervention Arm X Time X Moderator). This three-way interaction coefficient indicates whether change in average outcome varies by levels of the moderator while controlling for change in the control group. When a three-way interaction term is significant, we will re-estimate the models, stratified by level of the moderator, to generate strata-specific effect sizes. Using the gender model as an example, and significance set to *p* = 0.05, assuming ~ 50% of our child sample will be male, with our sample size of *n* = 30 male and *n* = 30 female children enrolled in Little Holy One, we will have 80% power to detect a medium (f = .24) difference in intervention effects between genders.

### Data collection methods

Data will be collected and managed in a REDCap database [49, 50]. All questionnaires are built in REDCap and data are directly entered by the participant or the evaluator. The evaluator will be trained on both the function of REDCap and the purpose and execution of each study instrument as well as open and sensitive interviewing methods.

The study schedule is designed to retain participants through all 12 intervention lessons and a range of evaluation time points. Gift card incentives will be given at each evaluation session. Study-related gifts and necessary supplies, such as smudging materials and notebooks at intervention lessons and food supplies and recipes at control lessons, will be provided. We have allocated one Head Start semester (total of 16 weeks) to complete the 12-week intervention or control session, providing an additional time to complete any make-up sessions and to account for any delays related to illness. Participants who discontinue or deviate from the intervention protocol will continue to complete the assessments as planned unless they withdrawal from the study. If a participant chooses to leave the study, they will not be provided with further intervention or control sessions. Enrolled participants will also be referred to appropriate resources, as needed, throughout the study.

### Statistical methods

#### Aim 1

The primary effectiveness aim will be assessed on a full intent-to-treat basis. The design incorporates longitudinal measurements that led to a nested data structure. For Aim 1 there are 5 repeated assessments (level-1) nested within children and caregivers (level-2) nested with 4 community health workers (level-3). Given the complexities of nested data, we will use mixed-effects regression models to analyze the data. Mixed-effects regression models are highly flexible and accommodate variability in the number and spacing of assessments within and across participants, continuous and discrete outcome distributions, varied patterns of change over time (e.g., quadratic), and phase-specific change (i.e., discontinuous or piecewise change [79, 81, 82]. To build our outcome models, we will first explore outcome trajectories graphically and descriptively evaluated to determine the best distribution for modeling using “spaghetti plots.” We will then iteratively build our estimation models using the approach described in Singer and Willett [83]. The first model will estimate the proportion of outcome variance at each level; the second model will add growth terms; the third model will add fixed-effect indicators to test for differences in baseline characteristics if necessary [83]. If there are differences in baseline characteristics between the intervention groups, these will be statistically controlled in models that follow. The fourth, and final, model will add the condition indicator (i.e., 0 = WLC, 1 = Little Holy One) and cross-level interaction effects between condition and level-1 terms.

#### Aim 2

We will again use mixed-effects regression models to analyze the outcomes. Mixed-effects regression models can evaluate phase-specific change while maintaining statistical power. This is essential for evaluating SCEDs due to the repeated measurements of the primary outcomes within each phase; and the interventions (i.e., delivery of cultural components) occurring at different time points within these phases. The key features of a SCED are that a) each participant will have a series of repeated measurements on the primary outcome; (b) the measurements occur across multiple experimental time periods, known as phases; and (c) each participant serves as its own comparison group as he/she progresses through the phases. This design is highly flexible and robust to unanticipated events (e.g., disruption in home life such as moving, etc.). Longitudinal assessments make it possible to straightforwardly model and control for these types of events by using time-varying indicators. Further, the use of multilevel models will partition the outcome variance into components specifically attributable to variability over time and across phases. Likewise, in a SCED everyone has their own starting value, yielding individual-specific outcome trajectories that are adjusted for unanticipated events occurring over time. As a result, it is possible to provide a strong evaluation of the impact of additional cultural components on individual trajectories of change while in the intervention.

A similar model building approach will be used including initially examining trajectories of session-by-session scores on the PSS, followed- by incremental model building like Aim 1. The final model will add the time-varying condition indicators (i.e., 0 = no cultural component; 1 = cultural component) and interaction effects between condition and the linear terms for change over time. This approach will create distinct sub-phases for each participant: before the component was delivered and after the component was delivered. Statistically, this modeling approach allows for determination of whether there is a shift in the overall level of the outcome or in the slope of the outcome following the delivery of the cultural component.

#### Aim 3

We will explore the bivariate associations between parent social network characteristics and suicide risk and opioid use. After a foundational understanding of the bivariate relationships is established, we will use generalized linear regression models to investigate the multivariate relationships between individual network characteristics on the outcomes of suicidal behaviors and opioid use. Additionally, we will assess the effect of Little Holy One on individuals’ social networks. The literature continues to support the use of social network measures as predictors of behavioral outcomes yet is less clear when it comes to the role behavioral interventions have on individuals’ social networks [75, 76]. We hypothesize that individuals that are randomized to the intervention group will see a significant positive change in their network composition compared to those in the control arm of the study due to the culturally salient nature of the content of the intervention arm. To assess this, we will use the pre-intervention data and the initial post-intervention data to assess changes in social networks over time between treatment groups.

### Intervention fidelity

To improve quality and adherence to intervention protocols, a session summary form will be completed by interventionists at the conclusion of each intervention/control session documenting what content was covered, how much time the visit lasted, any adverse event reporting or other concerns. All sessions will be audio-recorded and a random 20% will be listened to by the research coordinator on a continuous basis and rated for fidelity. Intervention fidelity is rated on 20 items, on a scale of 1 to 4, with 1 indicating the interventionist did not complete the requirement and 4 indicating the interventionist exceeded expectations. Concerns related to fidelity will be addressed in real time. Additionally, the on-site coordinator will complete quarterly observation checks with each interventionist to provide feedback on lesson delivery and support training for any necessary areas of improvement. The study Principal Investigator (PI) and Co-Investigator in charge of intervention training will work with the on-site project manager to provide immediate feedback, support, and booster training for interventionists if there are any concerns about fidelity adherence.

### Data monitoring and quality assurance

REDCap, hosted on secure virtual servers, provides forms for direct data entry that limit responses to appropriate data coding formats [49, 50]. REDCap also allows built-in quality checks during data collection, including range checks for entered values and warnings about questions left blank. The system also provides audit trails for tracking data entry, manipulation, import, and export. The data manager will also complete weekly data checks for quality and completeness using the REDCap data quality module to find issues with specific questions or instruments. Concerns about implementation of data collection will be addressed between the study coordinators, evaluators, and the data manager at weekly evaluation meetings.

### Safety of study participants

A Data Safety Monitoring Board (DSMB) will provide independent safety review and trial guidance during the ongoing study. Throughout the study period, the DSMB will review study processes and progress, adverse event data, and outcomes across intervention and control groups to judge whether the overall safety and feasibility of the trial remains acceptable. The DSMB may suggest appropriate courses of action to address general study safety issues which may arise. If warranted, the DSMB may recommend at any time that the entire protocol be suspended temporarily or terminated permanently. These recommendations will be directed to the study sponsor (National Institutes of Health) which has the responsibility to accept, reject or modify DSMB recommendations.

### Harms

The PI will assume responsibility of monitoring participants’ safety, including reporting any serious and unexpected Adverse Events (AEs). Any AEs will be categorized by investigators as ranging from mild to serious. Adverse Events include observed signs or participant disclosure of child abuse/neglect/domestic violence, acute substance intoxication, signs of substance abuse. Events that will be considered Serious Adverse Events (SAEs) include: any death of a study participant, a disability or incapacity which, in the opinion of the investigators, causes substantial disruption of a study participant’s ability to conduct normal life functions, hospitalization or extension of an existing hospitalization (excluding elective hospitalization for conditions unrelated to the study) and any intervention required to prevent one of the above outcomes. Serious and unexpected AEs will be reported within three business days to Johns Hopkins IRB, tribal IRBs and other tribal review entities, and the relevant NIH program officer. The PI will report actions taken by any of these groups in response to AE reports to each of the other groups in real time.

An Incidental Finding (IF) is a finding concerning an individual participant that has potential health importance and is discovered while conducting research but is beyond the aims of the study. As this is a behavioral/mental health study, most IFs that arise would be related to AEs. An IF outside of those necessitating mandatory reporting might be disclosure or discovery of a learning disability, disclosure of other risky behavior, but not immediately life-threatening (e.g., unprotected sex), disclosure of an illegal act, etc. Confidentiality will only be breached when someone is a danger to themselves or others; or they themselves are in immediate danger. Other disclosures are protected by an NIH Certificate of Confidentiality, which prevents any disclosures of participant information collected for research purposes, unless the participant provides consent for disclosure [84]. A resource card with health and mental health services will also be provided to all participants.

## Discussion

This study was designed to address the high prevalence of childhood trauma and related substance use and suicide behavior in Fort Peck Assiniboine and Sioux youth, by intervening with parents during a critical development stage for their child. Using a modular design approach [7], we selected and adapted four lessons based on content from *CETA,* four lessons based on content from *Family Spirit* and four lessons that aim to build on cultural strengths as health promotion and prevention content (i.e., the “cultural components”), which were co-created with our Tribal Advisory Board and key community leaders. The RCT with embedded SCED will allow us to simultaneously determine the effectiveness of a modular prevention program on caregiver and child outcomes and examine the added impact of specific cultural components. Our exploratory aims will allow us to examine social networks heterogeneity in overall effects, understand the relational factors that may increase both risk of suicide and opioid use and the relational characteristics of networks that act as protective factors for suicide and opioid use. Our study directly responds to the call for early trauma intervention and substance use and suicide prevention from Fort Peck Tribal Leaders and our Tribal Advisory Board.

### Limitations

There are several limitations to our study. First, most measures rely on self-report. To address this limitation, we added assessments to triangulate self-reported data, including parent and teacher reports. Second, including an active control may dilute the intervention impacts. However, community advisors stressed the importance of providing useful information to both groups, and nutrition lessons will steer clear of direct mental health promotion. Further, in low-resource settings such as the one in this study, utilizing an active control group is an ethical consideration. The control condition that was selected is both beneficial to the population—nutrition education in a community with a lack of food security and sovereignty—and for its minimal risk for confounding measure of primary and secondary intervention effects. Third, the Little Holy One curriculum has been developed through continuous consultation and advisement with a Tribal Advisory Board, resulting in lessons created for and adapted to a very specific target population. Due to this, results may have limited generalizability. That said, the other eight lessons on parenting, coping, and mental health promotion have been proven effective in their original form (before adaptation for this trial) in several other settings. Lastly, the currently planned follow-up is limited to 2 years, which may not be sufficient time to see the full impact of the intervention. Investigators plan to obtain funding in the future to follow study participants longitudinally into adolescence and young adulthood.

## Data Availability

The datasets generated and analyzed during the current study are not publicly available due to the tribal law that supports this research to respect their tribal sovereignty and confidentiality, but will be made available from the corresponding author on reasonable request in the most user-friendly, accessible, secure, and ethical format. We will take necessary steps to ensure we adhere to Fort Peck Tribal Law (Resolution #28–1744-2017) and NIH guidelines on sharing of data, in collaboration with our tribal partners.

## Abbreviations

ACE(s): Adverse childhood experience(s)
CETA: Common Elements Treatment Approach
RCT: Randomized control trial
SCED: Single-case experimental design
REDCap: Research Electronic Data Capture
DSMB: Data Safety Monitoring Board
AE: Adverse event
SAE: Serious adverse event
IF: Incidental finding
